# Supplementary material for: Content-rich biological network constructed by mining PubMed abstracts
Source: BMC Bioinformatics. 2004 Oct 8;5:147. doi: 10.1186/1471-2105-5-147 (PMC528731; doi:10.1186/1471-2105-5-147)
Supplement: Additional File 2 — The original results of the above study (non-essential files are deleted to keep the file size under the limit set by BMC bioinformatics). [file 1471-2105-5-147-S2.bz2 › chilibotAdditionalFile2/dip50/383ID10713102E9796/html/NTF3_NTRK3.html]

 


 **NTF3** and **NTRK3** 
  
Found 456 abstracts in PubMed, retrieved 50.  
 

 What does Google say? 
 PDF only 
| .edu only 

---

**Interactive relationship** (e.g. stimulation, inhibition, etc)

**Neutral relationship**- Rat cerebral endothelial cells express trk C  [ **NTRK3** ]  and are regulated by neurotrophin 3  [ **NTF3** ] .  Ref: 12745089 Biochem Biophys Res Commun, 2003
- The trophic synergism of NT3  [ **NTF3** ]  and BDNF may result from up regulation of TrkC  [ **NTRK3** ] .  Ref: 12009766 Exp Neurol, 2002
- Cells were either pretreated in vitro prior to transplantation with trkC  [ **NTRK3** ]  ligand neurotrophin 3  [ **NTF3** ]  NT 3  [ **NTF3** ]  to initiate differentiation or exposed to NT 3  [ **NTF3** ]  in vivo following transplantation via gelfoam or Oxycel.  Ref: 12075995 Cell Transplant, 2002
- NT 3  [ **NTF3** ]  mRNA was found in the cerebellar external granule layer and trkC  [ **NTRK3** ]  mRNA in the Purkinje layer of the cerebellar primordia.  Ref: 12106420 Eur J Neurosci< MedlineTA>Eur J Neurosci, 2002
- The embryonic Sertoli cells are the first testicular cells to differentiate and have been shown to express neurotropin 3 NT3  [ **NTF3** ] , which can act on high affinity trkC  [ **NTRK3** ]  receptors expressed on migrating mesonephros cells.  Ref: 12606390 Biol Reprod, 2003
- We also show that this processing, which does not occur in the TrkC  [ **NTRK3** ]  catalytic counterpart, is upregulated by NT 3  [ **NTF3** ]  and upon treatment with the tumor promoter 12 O tetradecanoylphorbol 13 acetate.  Ref: 12569366 Oncogene, 2003
- The results from in situ hybridization and immunostaining for TrkC  [ **NTRK3** ]  receptor revealed up regulation of the mRNA and protein by combining NT 3  [ **NTF3** ]  and BDNF.  Ref: 12009766 Exp Neurol, 2002
- New findings were RT PCR for neurotrophins and their receptors detected NT 3  [ **NTF3** ]  and NT 4 5 in alveolar macrophages, BDNF, NT 4 5, trkA, the truncated form of trkB, and trkC  [ **NTRK3** ]  in lung homogenate, but no trks in alveolar macrophages, immunohistochemistry for neurotrophin receptors detected TrkA in capillary cells, the truncated form of TrkB, and TrkC  [ **NTRK3** ]  in interstitial macrophages, immunoelectron microscopy for TrkC  [ **NTRK3** ]  revealed expression of TrkC  [ **NTRK3** ]  on the surface of interstitial macrophages, and in situ hybridization for neurotrophins detected BDNF in interstitial macrophages and alveolar type I cells, NT 3  [ **NTF3** ]  in alveolar macrophages, and NT 4 5 in alveolar and interstitial macrophages.  Ref: 12122447 Histochem Cell Biol, 2002
- Our results show that, following motor neuron deletion, more trkC  [ **NTRK3** ]  afferents project in cutaneous nerves on the deleted side of NT3  [ **NTF3** ]  treated embryos.  Ref: 12221005 Dev Biol, 2002
- It was found that peptidomimetic 2b has NT 3  [ **NTF3** ]  like neurotrophic activity in cell survival assays, selectively binds the NT 3  [ **NTF3** ]  receptor TrkC  [ **NTRK3** ] , and induces the tyrosine phosphorylation of the TrkC  [ **NTRK3** ]  receptor.  Ref: 12238916 J Med Chem, 2002
- Here we show that p62 interacts not only with TrkA, but also with TrkB and TrkC  [ **NTRK3** ] , which are the primary receptors for brain derived neurotrophic factor and neurotrophin 3  [ **NTF3** ] .  Ref: 12471037 J Biol Chem< MedlineTA>J Biol Chem, 2002
- Therefore, truncated trkC  [ **NTRK3** ]  plays a more important role in the development of this innervation than kinase forms of trkA or trkC  [ **NTRK3** ]  or of NT3  [ **NTF3** ] , the only known ligand for trkC  [ **NTRK3** ]  receptors.  Ref: 12117822 Development, 2002
- Nerve growth factor, brain derived neurotrophic factor and neurotrophin 3  [ **NTF3** ] , members of the neurotrophin family, bind to and activate TrkA, TrkB and TrkC  [ **NTRK3** ] , respectively, members of the Trk receptor tyrosine kinase family, to exert various effects including promotion of differentiation and survival, and regulation of synaptic plasticity in neuronal cells.  Ref: 12419535 Brain Res, 2002
- BDNF and NT 3  [ **NTF3** ]  mRNAs were expressed in the sensory epithelia of the cochlea and vestibule macula of the sacculus and utricle, and both trkB and trkC  [ **NTRK3** ]  mRNA were expressed in the spiral and vestibule ganglia innervating these sensory structures.  Ref: 12106420 Eur J Neurosci< MedlineTA>Eur J Neurosci, 2002
- The distinct distribution of trkA receptors on small neurons and trkC  [ **NTRK3** ]  receptors on large neurons in the dorsal root ganglion correlates with the dependence of these two classes of neurons on nerve growth factor and neurotrophin 3  [ **NTF3** ] , respectively, for survival during development.  Ref: 12603263 Eur J Neurosci, 2003
- NGF and NT 3  [ **NTF3** ]  mRNA were found in the iris, innervated by the sympathetic neurons of the superior cervical ganglion and sensory neurons from the trigeminal ganglion, which expressed both trk and trkC  [ **NTRK3** ]  mRNAs.  Ref: 12106420 Eur J Neurosci< MedlineTA>Eur J Neurosci, 2003
- ... TrkC  [ **NTRK3** ]  receptors are responsible for neurotrophin 3  [ **NTF3** ]  inhibition.  Ref: 12424382 Science, 2002
- Moreover, GM1 mediated TrkC  [ **NTRK3** ]  autophosphorylation was blocked by TrkC  [ **NTRK3** ]  IgG receptor bodies, further suggesting that GM1 activates TrkC  [ **NTRK3** ]  by inducing the release of neurotrophin 3  [ **NTF3** ] .  Ref: 12388556 J Biol Chem, 2002
- We also show that prolonged infusion of NT 3  [ **NTF3** ]  leads to a decrease in kindling induced Trk phosphorylation and a down regulation of the high affinity Trk receptors, TrkA and TrkC  [ **NTRK3** ] , suggesting an involvement of both cholinergic nerve growth factor receptors and hippocampal NT 3  [ **NTF3** ]  receptors in these effects.  Ref: 12453498 Neuroscience, 2002
- In addition, increased migration of trkC  [ **NTRK3** ]  overexpressing cells throughout the spinal gray matter was noted, particularly following in vivo NT 3  [ **NTF3** ]  exposure.  Ref: 12075995 Cell Transplant, 2002
- NT 3  [ **NTF3** ]  acts in an opposite fashion effecting a down regulation in BDNF expression in intact neurons and preventing reducing the injury associated increases in BDNF expression in both trkC  [ **NTRK3** ]  and nontrkC expressing subpopulations of injured neurons.  Ref: 12405958 Eur J Neurosci, 2002
- CONCLUSIONS It is known that brain derived neurotrophic factor and neurotrophin 3  [ **NTF3** ]  contribute to the differentiation of neuronal precursor cells, dendritic and axonal arborization, synaptic plasticity, and cellular hyperexcitability, so increased expression of trkB and trkC  [ **NTRK3** ]  may have a critical pathogenetic role in cytoskeletal abnormalities and epileptogenicity in dysplastic neurons of CD.  Ref: 12121325 Epilepsia, 2002

**Non-interactive relationship** (e.g. studied together, co-existance, homology, etc.)

- Neurotrophin 3  [ **NTF3** ]  and its receptor TrkC  [ **NTRK3** ]  were not modified compared to controls.  Ref: 12677448 Acta Neuropathol , 2003
- Changes in NT 3  [ **NTF3** ]  and TrkC  [ **NTRK3** ]  in the primary visual cortex of developing macaques.  Ref: 12352628 Neuroreport, 2002
- The role of the neurotrophin ligands, brain derived neurotrophic factor and neurotrophin 3  [ **NTF3** ] , and their respective tyrosine kinase receptors, TrkB and TrkC  [ **NTRK3** ] , in denervated taste buds is not known.  Ref: 12454994 J Comp Neurol, 2003
- Our results suggest that overexpression of BDNF, NT 3  [ **NTF3** ] , trkB and trkC  [ **NTRK3** ]  in motoneuron areas neighboring the site of mechanical compression may represent compensatory changes in response to the compromised neuronal function at the level of compression,.  Ref: 12774239 Acta Neuropathol Berl , 2003
- These embryos were further analyzed by using immunohistochemistry for trkC  [ **NTRK3** ]  a receptor for NT3  [ **NTF3** ]  retrograde and transganglionic labeling.  Ref: 12221005 Dev Biol, 2002
- The TrkC  [ **NTRK3** ]  receptor ligand, NT 3  [ **NTF3** ] , did not promote inhibitory synapse development in the absence of neuronal activity, and such cultures exhibited hyperactive cortical discharges.  Ref: 12647813 Histol Histopathol, 2003
- Mouse PTPRO mRNA was observed in neuron populations expressing TrkA, the high affinity nerve growth factor receptor, or TrkC  [ **NTRK3** ] , the neurotrophin 3  [ **NTF3** ]  receptor, and immunoreactive mPTPRO and TrkC  [ **NTRK3** ]  colocalized in large dorsal root ganglia proprioceptive neurons.  Ref: 12532410 J Comp Neurol, 2003
- To determine which of the neurotrophins NTs nerve growth factor NGF, brain derived neurotrophic factor BDNF, neurotrophin 3  [ **NTF3** ]  NT3  [ **NTF3** ] , and neurotrophin 4 5 NT4 and their receptors NTrs, TrkA, TrkB, TrkC  [ **NTRK3** ] , and p75, are present in the adult rat lacrimal gland.  Ref: 12882781 Invest Ophthalmol Vis Sci, 2003
- However, despite overlapping expression of TrkB and TrkC  [ **NTRK3** ]  in many sensory ganglia, brain derived neurotrophic factor and neurotrophin 3  [ **NTF3** ]  null mutant mice display selective losses in neuronal subpopulations.  Ref: 12620975 Development< MedlineTA>Development, 2003
- For this purpose, serial immunohistochemical and immunoblot analyses were performed for the expression levels of endogenous brain derived neurotrophic factor BDNF, neurotrophin NT 3  [ **NTF3** ] , and their receptors, trkB and trkC  [ **NTRK3** ]  in 24 tip toe walking Yoshimura mice twy twy aged 12 24 weeks.  Ref: 12774239 Acta Neuropathol Berl , 2003
- At the onset of Merkel ending development, at least one kinase lacking trkC  [ **NTRK3** ]  isoform is transiently expressed on all the follicle cells, while neurotrophin 3  [ **NTF3** ]  is transiently expressed only in the cells at the middle third of the follicle where the Merkel endings and cells develop.  Ref: 12117822 Development, 2002
- The combined trkC  [ **NTRK3** ]  expression and NT 3  [ **NTF3** ]  treatment appeared to reduce astrocytic differentiation of transplanted neural precursors.  Ref: 12075995 Cell Transplant, 2002
- Both treatments enhanced survival of trkC  [ **NTRK3** ]  overexpressing stem cells to nearly 100%, in comparison with approximately 30 50% when either NT 3  [ **NTF3** ]  or trkC  [ **NTRK3** ]  was omitted.  Ref: 12075995 Cell Transplant, 2002
- We identified a population of mature sympathetic neurons in which Ret, the receptor for glial cell line derived neurotrophic factor GDNF, is coexpressed with the neurotrophin 3  [ **NTF3** ]  NT3  [ **NTF3** ]  receptor TrkC  [ **NTRK3** ]  and choline acetyltransferase.  Ref: 11988020 Mol Cell Neurosci, 2002
- NGF, NT 3  [ **NTF3** ]  and TrkC  [ **NTRK3** ]  like immunoreactivity were never detected.  Ref: 12090394 J Anat, 2002
- the numbers of neurons expressing the NT 3  [ **NTF3** ]  high affinity receptor trk C  [ **NTRK3** ]  were unchanged.  Ref: 12598727 Neuroreport, 2003
- In contrast, the expression levels of BDNF, NT 3  [ **NTF3** ] , trkB and trkC  [ **NTRK3** ]  were significantly higher at the rostral and caudal sites immediately adjacent to the maximal compression site.  Ref: 12774239 Acta Neuropathol Berl , 2003
- Because neurotrophin 3  [ **NTF3** ]  NT 3  [ **NTF3** ]  is specifically produced by motoneurons and because interneurons express the NT 3  [ **NTF3** ]  high affinity receptor trkC  [ **NTRK3** ] , we examined the role of NT 3  [ **NTF3** ]  in the survival of PAX2 IR interneurons.  Ref: 12388583 J Neurosci, 2002
- In situ hybridization analysis of cells expressing messenger RNAs mRNAs for the neurotrophins nerve growth factor NGF, brain derived neurotrophic factor BDNF and neurotrophin 3  [ **NTF3** ]  NT 3  [ **NTF3** ]  and their high affinity receptors trk, trkB and trkC  [ **NTRK3** ]  in the rat embryo revealed a complex but specific expression pattern for each of these mRNAs.  Ref: 12106420 Eur J Neurosci< MedlineTA>Eur J Neurosci, 2002
- Cultured cortical progenitors express BDNF and NT 3  [ **NTF3** ] , as well as their receptors TrkB tyrosine kinase receptor B and TrkC  [ **NTRK3** ] .  Ref: 12832539 J Neurosci, 2003
- These cells express aberrant levels of functional p75 and TrkC  [ **NTRK3** ] , the putative high affinity receptor for the neurotrophin NT 3  [ **NTF3** ] .  Ref: 12616526 J Cell Biochem, 2003
- Consistent with a trkB.FL mediated mechanism, the i.t. administration of another trkB ligand, neurotrophin 4 5, also produces hyperalgesia while the trkC  [ **NTRK3** ]  agonist neurotrophin 3  [ **NTF3** ] , which weakly cross reacts with trkB, has little effect.  Ref: 12435470 Pain, 2002
- In the present study, we measured the levels of neurotrophin 3  [ **NTF3** ]  NT 3  [ **NTF3** ]  protein and mRNA by ELISA and quantitative RT PCR, and examined TrkC  [ **NTRK3** ]  immunoreactive structures using immunohistochemical method.  Ref: 12352628 Neuroreport, 2002
- In the presence of NT 3  [ **NTF3** ] , TrkC  [ **NTRK3** ]  phosphorylation and protein expression decreased significantly while activation of the IGF IR was maintained in the pro OLs in the presence of IGF I.  Ref: 12139923 Mol Cell Neurosci, 2002
- In single and double labeled immunofluorescent experiments, 09 taste bud sections from 13 hamsters were examined for immunopositive gemmal cells or nerve fibers using antibodies to BDNF and NT 3  [ **NTF3** ] , their respective receptors TrkB and TrkC  [ **NTRK3** ] , and the neural marker ubiquitin c terminal hydrolase L 1 protein gene product 9.5.  Ref: 12454993 J Comp Neurol, 2003
- We studied the interactions of neurotrophin 3  [ **NTF3** ]  NT3  [ **NTF3** ]  with brain derived neurotrophic factor BDNF, fibroblast growth factor 2 FGF 2, and their effects on tyrosine kinase C TrkC  [ **NTRK3** ]  expression during cochlear ganglion development.  Ref: 12009766 Exp Neurol, 2002
- Weak staining of NT3  [ **NTF3** ]  and TrkC  [ **NTRK3** ]  was noted in the suprabasal layers of corneal and limbal epithelia but was negative in the stratified HLE in nude mice.  Ref: 11923238 Invest Ophthalmol Vis Sci, 2002
- In this work the expression and anatomical localization of NGF, BDNF and NT 3  [ **NTF3** ]  and of corresponding high affinity receptors TrkA, TrkB full length and truncated TR isoforms, TrkC  [ **NTRK3** ]  and of the low affinity p75 receptor were assessed in surgical samples from adult human lung by reverse transcriptase polymerase chain reaction RT PCR, Western blot and immunohistochemistry.  Ref: 12791675 Am J Respir Cell Mol Biol< MedlineTA>Am J Respir Cell Mol Biol, 2002
- The neurotrophins brain derived neurotrophic factor and neurotrophin 3  [ **NTF3** ] , as well as their respective tyrosine kinase receptors, TrkB and TrkC  [ **NTRK3** ] , influence peripheral target cell innervation, survival, and proliferation.  Ref: 12454993 J Comp Neurol, 2003
- METHODS Since the biologic function of NT 3  [ **NTF3** ]  is mediated primarily through Trk C  [ **NTRK3** ] , we investigated Trk C  [ **NTRK3** ]  immunoexpression on paraffin sections of 10 compound nevi and 63 melanomas.  Ref: 12753172 J Cutan Pathol, 2003
- Down regulation of NT 3  [ **NTF3** ]  and BDGF was the most significant, and TrkC  [ **NTRK3** ]  down regulation level was the lowest in receptors Trk family.  Ref: 12854135 World J Gastroenterol, 2003
- Because TrkC  [ **NTRK3** ]  like IR in bud cells is absent, and sparse in fibers intragemmally and perigemmally, NT 3  [ **NTF3** ]  may also bind to bud cell TrkB so as to sustain fungiform gemmal cell viability post denervation.  Ref: 12454994 J Comp Neurol, 2003
- Immunoreactivities for BDNF, NT 3  [ **NTF3** ] , trkB and trkC  [ **NTRK3** ]  were preferentially localized in the gray matter, particularly in the anterior horn cells.  Ref: 12774239 Acta Neuropathol Berl , 2003
- Western blot analysis confirmed these results, showing that the lacrimal gland expresses NGF, BDNF, NT3  [ **NTF3** ] , and NT4 as well as the NTrs TrkA, TrkB, and TrkC  [ **NTRK3** ]  and the p75 protein.  Ref: 12882781 Invest Ophthalmol Vis Sci, 2003
- RT PCR showed positive bands at the appropriate sizes for NGF, BDNF, NT3  [ **NTF3** ] , and NT4, and for the receptors TrkA, TrkB, TrkC  [ **NTRK3** ] , and p75.  Ref: 12882781 Invest Ophthalmol Vis Sci, 2003
- In the current study, we tested whether submaximal concentrations of NT 3  [ **NTF3** ]  would maintain receptor tyrosine kinase TrkC  [ **NTRK3** ]  activation and Akt phosphorylation and thus promote long term survival of the pro OL against glutamate.  Ref: 12666655 Dev Neurosci, 2002
- We found that 1 trk C  [ **NTRK3** ] , a receptor for neurotrophin 3  [ **NTF3** ]  NT 3  [ **NTF3** ] , is dominantly expressed in RCEC, but trk B, a receptor for brain derived neurotrophic factor, is dominantly expressed in RAEC.  Ref: 12745089 Biochem Biophys Res Commun, 2003
- This study shows that, in addition to alternative splicing, ectodomain shedding represents a novel means of regulating TrkC  [ **NTRK3** ]  receptor signaling, and consequently NT 3  [ **NTF3** ]  biological effects on target cells.  Ref: 12569366 Oncogene, 2003
- Our results demonstrate that NT 3  [ **NTF3** ]  at any concentration sufficient to activate the TrkC  [ **NTRK3** ]  receptor results in a transient phosphorylation of the receptor and of Akt due, in part, to downregulation of the Trk receptor.  Ref: 12666655 Dev Neurosci, 2002
- We have used immunofluorescence techniques with a panel of antibodies against known neurotrophin receptors trk A, trk B, trk C  [ **NTRK3** ] , p75NTR to map the locations of these receptors in the developing neuromuscular system of mice from our neurotrophin 3  [ **NTF3** ]  NT 3  [ **NTF3** ]  knockout colony.  Ref: 12141445 Int J Dev Biol, 2002
